# Factors influencing salivary lactate dehydrogenase levels in oral squamous cell carcinoma and oral potentially malignant disorders

**DOI:** 10.3389/froh.2024.1525936

**Published:** 2025-01-07

**Authors:** Rosa María López-Pintor, José González-Serrano, Carmen Vallina, Katerina Ivaylova Serkedzhieva, Leire Virto, Paula Nuevo, Vito Carlo Alberto Caponio, Margarita Iniesta, Tania Rodríguez Santamarta, Paloma Lequerica Fernández, Óscar Iglesias Velázquez, Gonzalo Hernández, Juan Carlos de Vicente

**Affiliations:** ^1^ Department of Dental Clinical Specialties, Faculty of Dentistry, Complutense University, Madrid, Spain; ^2^ ORALMED Research Group, Department of Dental Clinical Specialties, School of Dentistry, Complutense University, Madrid, Spain; ^3^ Department of Anatomy and Embryology, Faculty of Optics and Optometry, Complutense University, Madrid, Spain; ^4^ Research Laboratory, Faculty of Dentistry, Complutense University, Madrid, Spain; ^5^ Department of Clinical and Experimental Medicine, University of Foggia, Foggia, Italy; ^6^ Department of Oral and Maxillofacial Surgery, Hospital Universitario Central de Asturias (HUCA), Oviedo, Spain; ^7^ Department of Biochemistry, Hospital Universitario Central de Asturias (HUCA), Oviedo, Spain

**Keywords:** L-Lactate dehydrogenase, saliva, biomarkers, oral squamous cell carcinoma, mouth neoplasms, oral leukoplakia, oral lichen planus, precancerous conditions

## Abstract

**Introduction:**

Salivary Lactate Dehydrogenase (sLDH) levels seem to be higher in patients with Oral Squamous Cell Carcinoma (OSCC) and Oral Potentially Malignant Disorders (OPMD) than a control group (CG).

**Methods:**

Case-control study. Patients with OPMD [oral leukoplakia (OL) and oral lichen planus (OLP)] and OSCC who attended two services in Spain were selected. sLDH in saliva was measured. Epidemiological, periodontal and specific variables related to OPMD and OSCC were collected.

**Results:**

A total of 92 patients were included: 12 with OSCC, 51 with OPMD (17 OL and 34 OLP), and 29 controls. sLDH values were higher in the OSCC, followed by the OPMD and CG groups, although no significant differences were observed. In the OSCC group, larger tumor size was associated with higher sLDH levels. In the OLP group, sLDH values were higher in patients with symptomatic lesions than in patients with only white lesions, but not significantly. No associations were observed between sLDH and the type of OL (homogeneous vs. non-homogeneous) and the degree of dysplasia. When analyzing periodontal variables among OSCC, OPMD and CG, periodontal probing depth (PPD) and bleeding on probing were significantly higher in the OSCC group, while the plaque index was higher in OPMD patients. The linear regression model for sLDH in the total group identified age and PPD as significant predictors of sLDH levels.

**Discussion:**

Although sLDH values were higher in OSCC and OPMD patients than in a CG, the results do not support the use of sLDH as a reliable prognostic biomarker of malignancy. Future studies need to consider other factors that may influence sLDH levels, such as age and periodontal status.

## Introduction

Cancer of the oral cavity and lip is a relevant disease that ranks 16th in global cancer incidence (1, 2), with oral squamous cell carcinoma (OSCC) being the most prevalent. The diagnosis of OSCC is more frequent in men over 45 years of age, although the incidence in women is increasing. OSCC risk factors include tobacco, areca nut, betel quid, and heavy alcohol consumption, among others (1–5). Women with OSCC tend to have fewer classic risk factors (tobacco and alcohol) and better survival rates than men (6). Socioeconomic factors, including low income and education, also increase the risk of oral cancer (7). OSCC is often diagnosed at advanced stages, which decreases survival rate (5, 7). Patients with Oral Potentially Malignant Disorders (OPMD) have a higher susceptibility to OSCC than those without these disorders (7, 8). Therefore, it would be helpful to have biomarkers to identify high-risk OPMD patients, which could help in the early diagnosis of OSCC (9, 10).

A biomarker is a characteristic that can be objectively measured and evaluated as an indicator of normal biological processes, pathological processes, or pharmacological responses to a therapeutic intervention (11). These biomarkers can be detected in saliva. For years, efforts have been made to try to discover biomarkers that can help in the diagnosis of OPMD and OSCC, and to predict which OPMD can evolve into OSCC (12). Saliva can be a fluid in which these biomarkers can be detected, as these lesions are found in the oral mucosa. Saliva collection is a simple and minimally invasive technique that can be performed repeatedly at different time intervals, which offers advantages over other means such as serum or tissues (13, 14). One such biomarker is salivary lactate dehydrogenase (sLDH), an enzyme involved in aerobic glycolysis by catalyzing the pyruvate to lactate reaction. This enzyme is normally found in the cytoplasm of most cells in the human body, but when oxidative stress is present, it is released outside the cell. Therefore, increased LDH levels might suggest tissue damage and cell necrosis (15–17), as seen in OSCC and OPMD patients. Studies have hypothesized that LDH could be a useful biomarker for predicting malignancy in OPMD (17, 18).

Numerous studies have shown that sLDH levels are higher in OSCC patients compared to controls (9, 15, 18–28). Studies on oral leukoplakia (OL) found higher sLDH levels in these patients compared to a control group (CG) although sLDH levels were lower than patients with OSCC (15, 18–20, 29, 30). Similarly, oral submucous fibrosis (OSMF) patients exhibited significantly higher sLDH values compared to a CG (25, 31) but lower values compared to OSCC patients (25). Oral lichen planus (OLP) is currently considered an OPMD because it has been observed that it can evolve to OSCC over time (8). However, its malignant potential varies widely depending on the diagnostic criteria used, with malignant rates ranging from 0.2% to 1.43% (32, 33). Risk factors for malignant transformation of OLP include smoking, alcohol consumption, hepatitis C infection, atrophic and erosive forms of OLP, lesions located on the tongue and epithelial dysplasia (32, 33).

To date, only one previous study (27) has evaluated sLDH levels in patients with OLP and oral lichenoid reactions. This study found that sLDH levels were higher in these patients than in a CG, but lower than in the OSCC group. In general, prior studies and meta-analyses (17, 34, 35) indicated that sLDH levels are higher in OSCC patients, followed by OPMD patients, and lower in healthy patients. These findings suggest that sLDH detection could help assess progression to OSCC, although no definitive cut-off values for OSCC and OPMD have been established (17, 35). Notably, all previous studies were conducted in Asia, limiting their global applicability. In fact, in Europe, OSMF is rare, while OLP is more prevalent (36), and only one previous study has been conducted on this OPMD (27).

Smoking also influences sLDH levels; smokers had higher levels than non-smokers (37, 38). While the carcinogenic effect of tobacco raises sLDH levels in smokers and gutkha chewers (39), several investigations found that smoking-only patients have lower sLDH levels than OSCC and OL patients (30, 40).

On the other hand, research has also shown that sLDH levels are higher in patients with periodontitis than in those without periodontitis or gingivitis (41). sLDH levels increases with the severity of periodontitis (42), and correlate positively with periodontal indicators such as probing depth (PPD), plaque index (PI), and bleeding on probing (BOP) (43, 44). Previous studies showed that sLDH levels decreased after supra- and subgingival mechanical instrumentation, (45, 46) as well as periodontal indicators (46). Age-related increases in sLDH levels have also been observed (41, 43), potentially due to increased prevalence of systemic diseases, reduced salivary flow, and changes in oral biofilm with aging (41).

In view of the above, further studies are needed in non-Asian countries to examine how the above mentioned factors (OSCC, OPMD, smoking, periodontal status, and age) influence sLDH levels, validating its role as reliable biomarker. Therefore, this study aimed to evaluate whether total sLDH levels are elevated in patients with OSCC and OPMD (specifically OLP and OL) compared to a CG without these oral disorders and to identify those clinic and pathological factors with impact on sLDH concentration in these patients.

## Materials and methods

### Study design

This is an observational case-control study. The study was conducted in accordance with the Declaration of Helsinki and received approval from the Ethics Committees of the Hospital Clínico San Carlos (no. 21/020-E) and Principado de Asturias (no. 2021.030). The study also followed the guidelines established by the Strengthening the Reporting of Observational Studies in Epidemiology (STROBE) Statement (https://www.strobe-statement.org). Prior to their participation in the study, patients read the information sheet and signed the informed consent form.

### Participants

Sixty-three patients with OPMD and OSCC were included in this study. OPMD (*n* = 51) and OSCC (*n* = 12) patients who attended to the Oral Medicine Specialization at Universidad Complutense of Madrid and the Maxillofacial Surgery Service at the Hospital Universitario Central de Asturias, between January 2022 and June 2023, were included in the study.

OSCC and OPMD patients had to meet the following inclusion criteria: (a) be over 18 years of age; (b) be clinically and histologically diagnosed with OLP (47), OL (8) or OSCC. The exclusion criteria were as follow: (a) being previously diagnosed of any cancer and/or having received oncologic treatment for it; (b) having started OSCC oncologic treatment; (b) patients under treatment with topical corticosteroids in the previous 4 weeks or systemic corticosteroids in the previous 8 weeks; (c) patients with diseases that may alter sLDH values (e.g., acute myocardial infarction in the 6 months before attendance, liver disease, renal disease or muscular dystrophy); (d) pregnant or breastfeeding women; (e) patients who did not accept to sign the informed consent form; and (f) patients who did not follow the instructions prior to saliva collection.

The CG (*n* = 29) consisted of patients similar in age and sex to the patients with OSCC and OPMDs. These patients were recruited from the General Dental Clinic of the Faculty of Dentistry at Complutense University. New patients and those attending for dental checkups were invited to participate in the study. The CG patients had to meet the following inclusion criteria: (a) be older than 18 years of age; and (b) not suffer from oral lesions of OPMD and OSCC. The exclusion criteria were the same for patients in both study groups.

### Salivary flow collection

Unstimulated whole saliva (UWS) was collected between 8:00 a.m. and 11:00 a.m. after asking participants not to brush their teeth, eat, drink, or smoke 90 min before the appointment. Patients were seated in a relaxing place, and they were asked to push the head forward to accumulate saliva and then drop it into a sterile container without making any effort with the mouth. Salivary flow was collected for 10 min (48).

### Saliva preservation protocol and determination of LDH levels

The collected saliva was centrifuged at 1,160 g for 20 min to separate the supernatant, containing soluble components, from the cellular debris. The supernatants were then stored at −80°C until analysis. The concentration of sLDHs was measured using the Lactate Dehydrogenase Assay Kit Colorimetric (ab 102,526) from Abcam (Cambridge, United Kingdom). sLDH concentration was analyzed spectrophotometrically at a wavelength of 450 nm. The results obtained were extrapolated to a standard line to obtain LDH concentrations in international milliunits per milliliter (mU/ml), which is equivalent to IU/L.

### Clinicopathological features

Clinicopathological features were obtained from the patient's medical history. The following clinical variables were registered for each patient: age, sex, tobacco (yes/no and number of cigarettes/day), alcohol (yes/no and dose/day), systemic diseases and drugs received.

In patients with OPMD and OSCC, specific variables were also collected:

– OSCC: location and TNM classification. Histological differentiation was also collected after histological analysis following surgery (7, 49).
– OLP: location and type of lesions (reticular, papular, atrophic and/or erosive, or bullous) (50). Subsequently, they were also classified into white or atrophic erosive lesions.
– OL: location, type of OL (homogeneous or non-homogeneous) (8) and degree of dysplasia (no dysplasia, mild, moderate, or severe dysplasia) (51). Three-tiered grading of oral epithelial dysplasia was used, since binary grading is not yet validated (51).

The collection of periodontal variables was performed by two trained and calibrated clinicians on all teeth present. The examiners were previously calibrated at the beginning of the study by evaluating 6 patients and recording the variables twice consecutively, with a minimum interval of 60 min.

Periodontal variables were collected on the day the patient attended the clinic for the saliva collection and thereafter. The periodontal parameters collected were PPD, PI, and BOP. These measurements were performed at 6 sites per tooth (mesio-vestibular, vestibular, disto-vestibular, mesio-lingual/mesio-palatal, lingual/palatal, disto-lingual/disto-palatal). A CPC15 periodontal probe (HuFriedy®, Chicago, IL, USA) was used.

In the case of the periodontal clinical variable PPD, the values of all the sites were averaged to obtain the final value for each patient. In the case of the categorical periodontal variables BOP and PI, the percentage of total sites with bleeding or plaque was calculated.

### Sample size

Sample size was determined using data from a previous study comparing sLDH levels in OSCC, OL, and control patients (15). An alpha risk of 0.05 and a power of 90% were assumed, with a difference of at least 30 IU/L in sLDH concentration between the OSCC group and the CG. Applying the sample size formula for the comparison of two means *n* = (Zα/2 + Zβ)2 *2*σ2/d2 (52), at least 11 participants per group were necessary.

### Statistical analysis

The statistical analysis included descriptive statistics, categorical variables are shown as number and percentage and quantitative variables as mean (standard deviation). Normality tests of quantitative variables were performed using the Shapiro–Wilk test. Categorical variables were compared between groups using the Chi-square test. The association between categorical variables and sLDH values when there were two groups was performed using the *U*-Mann–Whitney test. When there were more than two groups, they were analyzed with the Kruskal–Wallis test applying the Bonferroni correction for multiple comparisons. Correlations between two quantitative variables were analyzed using the Spearman's correlation. A Multiple linear regression was performed to assess the factors influencing sLDH values. Variables that had obtained statistical significance in the bivariate analysis were included in the regression model. Values with a *p* ≤ 0.05 were considered statistically significant. Statistical analysis was performed using the IBM® SPSS® Statistics version 29.0 (IBM Corp., Armonk, N.Y., USA).

## Results

This study evaluated 92 patients: 12 patients with OSCC, 51 with OPMD (17 with OL and 34 with OLP), and 29 controls (Table 1). When the patients were classified into three groups (OSCC, OPMD and CG), the three groups were similar in sex, age, smoking, alcoholic habits, and UWS flow rate (ml/min). However, when both types of OPMD were considered separately (OSCC, OLP, OL and CG), differences were observed in smoking habits, number of cigarettes consumed per day, and UWS (ml/min). OL group had more smokers, and cigarette consumption differed significantly between the OLP and OL groups (*p* = 0.027). UWS (ml/min) was also higher in the OL group, although no significant differences were observed between groups.

**Table 1:** Variables in the three groups of patients included (OSCC, OPMD and CG). The results of the variables in the different types of OPMD can also be observed separately. The results of the categorical variables are shown in number and proportion (%) and the quantitative variables in mean (standard deviation).

	OSCC	OPMD			CG *n* = 29	*p* OSCC, OPMD, CG	*p* OSCC, OLP, OL, CG
		OLP	OL	OPMD total			
Number of cases and % of total included patients	12 (13.04%)	34 (36.96%)	17 (18.48%)	51 (55.44%)	29 (31.52%)		
Age	69 (12.87)	64.47 (9.99)	65 (11.45)	64.65 (10.39)	59.83 (13.82)	0.21^a^	0.37^a^
Sex							
Male	4 (33.3%)	9 (26.5%)	7 (41.2%)	16 (31.4%)	12 (41.4%)	0.66^b^	0.59^b^
Female	8 (66.7%)	25 (73.5%)	10 (58.8%)	35 (68.6%)	17 (58.6%)		
Smoking habit (yes)	4 (33.3%)	3 (8.8%)	8 (47.1%)	11 (22.6%)	8 (27.6%)	0.65^b^	0.021^b^
Number of cigarettes/day	6.67 (9.85)	0.53 (2.61)	6.24 (8.36)	2.43 (5.85)	3.52 (7.46)	0.28^a^	0.014^a^
Alcohol habit (yes)	3 (25%)	6 (17.6%)	3 (17.6%)	9 (17.6%)	3 (10.3%)	0.47^b^	0.68^b^
Number of doses* of alcohol/day	1.25 (2.38)	0.26 (0.71)	0.29 (0.77)	0.27 (0.72)	0.07 (0.26)	0.2^a^	0.35^a^
PPD	4.38 (0.76)	2.73 (0.49)	2.92 (0.39)	2.79 (0.53)	2.79 (0.26)	0.05^a^	0.097^a^
BOP (%)	70 (13.62)	53.70 (19.43)	45.26 (24.33)	51.06 (21.19)	33.68 (18.91)	<0.001^a^	<0.001^a^
PI (%)	47.40 (39.60)	74.82 (18.17)	56.33 (27.59)	69.04 (22.94)	51.35 (29.76)	0.03^a^	0.008^a^
sLDH (IU/L)	179.01 (140.64)	137.88 (112.11)	84.52 (66.22)	120.09 (101.71)	110.54 (85.23)	0.43^a^	0.1^a^

OSCC, oral squamous cell carcinoma; OLP, oral lichen planus; OL, oral leukoplakia; OPMD, oral potentially malignant disorders; CG, control group; PPD, periodontal probing depth; BOP, bleeding on probing; PI, plaque index; sLDH, salivary lactate dehydrogenase.

* It was considered a dose of alcohol: a glass of wine, a beer or a spirit.

Kruskal–Wallis test (^a^) was applied for quantitative variables and the Chi-square test (^b^) for categorical variables.

Significant differences in periodontal variables were observed across the three groups (OSCC, OPMD and CG). PPD and BOP were higher in the OSCC group, while PI was higher in OPMD patients. A significant difference was observed in PPD between the OSCC and OPMD groups (*p* = 0.045). For BOP, significant differences were observed between the OPMD and CG groups (*p* = 0.003) and between the OSCC and CG group (*p* = 0.002). In terms of PI, a significant difference was observed between OPMD and CG (*p* = 0.037). Considering the four groups of patients (OSCC, OLP, OL and CG), BOP was higher in OSCC patients and PI in OLP patients. Significant differences in BOP were observed between the OLP and CG (*p* = 0.002) and between the OSCC and CG (*p* = 0.003). Significant differences in PI were only observed between OLP and CG (*p* = 0.01).

The sLDH values were higher in the OSCC group, followed by OPMD and CG groups, but differences among the groups were not significant. Within OPMD, OLP patients had higher sLDH levels than OL patients, although not significantly (*p* = 0.1).

### OSCC group

A total of 12 OSCC were included in the study: 5 located on the lateral border of the tongue, 4 on the floor of the mouth, 2 on the ventral side of the tongue, 2 on the alveolar ridge, one on the hard palate and one on the buccal mucosa. When analyzing the TNM classification of the included OSCC (Table 2), sLDH values increased with a higher value of T or size of the tumor. A statistically significant difference was observed between T3 and T4 tumors compared with T1 and T2 ones (*p* = 0.017), being the sLDH values higher in more locally extended tumors. Similarly, patients harboring neck lymph node metastasis showed higher sLDH values than patients without neck metastasis, although these differences were not statistically significant. Since all patients did not have distant metastases, sLDH values could not be analyzed according to this variable.

**Table 2:** Specific variables of OSCC, OLP and OL lesions and their association with sLDH values.

Group and variables	*n* (%)	sLDH [mean (SD)]	*p*
OSCC (*n* = 12)			
TNM classification			
Size of the tumor (T)			
T1	4 (33.3%)	75.67 (44.9)	0.082
T2	4 (33.3%)	151.76 (139.01)	
T3	2 (16.7%)	210.06 (6.72)	
T4	2 (16.7%)	409.11 (0.043)	
Lymph nodes (*N*)			
N0	7 (58.3%)	143.06 (111.05)	0.40
N1	2 (16.7%)	241.96 (236.33)	
N2	2 (16.7%)	126.78 (111.06)	
N3	1 (8.3%)	409.14	
Size of the tumor (T)			
T1 + 2	8 (66.7%)	113.71 (103.92)	0.017
T3 + 4	4 (33.3%)	309.58 (114.98)	
Lymph nodes (*N*)			
N0	7 (58.3%)	143.06 (111.05)	0.37
N+	5 (41.7%)	229.32 (174.55)	
OSCC differentiation			
Well differentiation	8 (66.6%)	180.12 (134.76)	0.77
Moderate differentiation	3 (24.9%)	211.35 (122.02)	
Poor differentiation	1 (8.3%)	205.31	
OSCC differentiation			
Well differentiation	8 (66.7%)	180.12 (134.76)	0.73
Moderate-poorly differentiation	5 (33.3%)	176.77 (173.71)	
OLP (*n* = 34)			
Type of OLP lesions			
White lesions (reticular or plaque)	28 (82.35%)	131.23 (107.19)	0.44
Erosive and/or ulcerative	6 (17.64%)	168.89 (139.7)	
OL (*n* = 17)			
Type of OL			
Homogeneous	14 (82.4%)	90.40 (71.58)	0.43
Non-homogeneous	3 (17.6%)	57.04 (20.19)	
Dysplasia			
No dysplasia	15 (88.24%)	88.35 (69.39)	0.62
Presence of dysplasia	2 (11.76%)	55.72 (29.70)	
Mild	2 (100%)	55.72 (29.70)	
Moderate	0	–	
Severe	0	–	

Chi-square test was applied for categorical variables and Kruskal–Wallis test or *U*-Mann Whitney test for quantitative variables. OSCC, oral squamous cell carcinoma; OLP, oral lichen planus; OL, oral leukoplakia; OPMD, oral potentially malignant disorders; TNM, TNM staging system for tumor, node, and metastasis.

Regarding OSCC differentiation, sLDH values of the well-differentiated were lower than those of the moderately differentiated. However, sLDH values of the poorly differentiated were higher than that of the moderately differentiated. Moreover, no significant differences in sLDH values were observed among the three groups.

### OLP group

Of the 34 OLP patients, 28 had white lesions (plaque and/or reticular lesions) and 6 had erosive and/or ulcerative lesions. sLDH values were higher in the group with erosive and/or ulcerative lesions than in the group with white lesions, but without statistical significance (Table 2).

### OL group

Of the 17 OL patients included, 14 had homogeneous lesions and 3 non-homogeneous. The sLDH values of the non-homogeneous group were not significantly higher than in the homogeneous ones (Table 2).

Of the OL patients included, 15 had no dysplasia and 2 had mild dysplasia, but the sLDH values did not differ significantly between these groups.

### Associations and correlations between variables and the sLDH values in the total group of patients

Smokers (*n* = 23) had lower sLDH values than non-smokers (*n* = 69) [101.26 (93.71)] vs. 132.6 (106.44)), although the difference was not statistically significant (*p* = 0.09).

In contrast, alcohol-consuming patients had higher sLDH levels than non-alcohol-consumers [132.99 (120.49) vs. 123.16 (101.05)], but this was also not significant (*p* = 0.98).

Regarding sex, women (*n* = 60) had higher sLDH values than men (*n* = 32) [134.63 (106.79) vs. 106.27 (96.86)]; however, this difference was not statistically significant (*p* = 0.69).

No significant correlations were observed between sLDH levels and tobacco dose (*r* = −0.17; *p* = 0.104) or between sLDH levels and alcohol dose (*r* = 0.003; *p* = 0.97). However, sLDH correlated positively with age (*r* = 0.28; *p* = 0.007), although the correlation was weak.

Finally, periodontal variables, specifically PPD (*r* = 0.26; *p* = 0.02) and BOP (*r* = 0.325; *p* = 0.003), correlated positively and significantly with the sLDH values, but these correlations were also weak. However, the correlation with PI was practically null and did not obtain statistical significance (*r* = 0.092; *p* = 0.41).

### Multiple linear regression

The linear regression model for sLDH included age, PI, PPD, and BOP (see Table 3). The model explained 27% of the variance (F = 7.06, *p* < 0.001, *R*^2^ = 0.27). Only age and PPD were significant predictors of sLDH, with higher age and PPD associated with a higher sLDH value.

**Table 3:** Multiple linear regression results for sLDH.

Variables	Coefficient B (SD)	95% confidence interval		*p*
		Lower limit	Upper limit	
Age (years)	2.60 (0.91)	0.78	4.43	0.006
PI	−0.58 (0.48)	−1.54	0.38	0.23
PPD	43.35 (13.98)	15.50	71.19	0.003
BOP	0.95 (0.62)	−0.29	2.19	0.13

PPD, periodontal probing depth; BOP, bleeding on probing; PI, plaque index.

## Discussion

The present study aimed to evaluate whether total sLDH levels were higher in OSCC and OPMD patients than in a CG without these oral disorders. The study also aimed to assess which factors influence sLDH values in these patients. While the mean sLDH values were higher in OSCC than in OPMD patients and CG, the differences were not statistically different. Regarding the factors influencing the sLDH values, higher PPD, a periodontitis-related variable, and older age were associated with higher sLDH values.

Salivary biomarker detection can be repeated periodically because it is a minimally invasive technique. These biomarkers could be a diagnostic tool to assess the malignant potential of OPMD (14). One of these salivary biomarkers is sLDH (14, 17). LDH is present in the epithelial cells of the oral mucosa. When oxidative stress occurs and cell necrosis increases, as may be the case in OSCC and OPMD, LDH can be released extracellularly and detected in saliva. The cells involved in these processes need more energy and therefore depend on anaerobic processes of glycolysis (17, 30, 50). In the present study, no significant differences in sLDH values were observed between patients with OSCC, OPMD and CG, but the results follow the trend of previous studies (9, 15, 17–28, 31, 50).

All previous studies on this topic have been conducted in Asia. The risk habits of these patients and those in our study are different and may have influenced the results. In Europe, cigarette smoking is common but it is unclear whether cigarette smoking influences sLDH levels, and studies in patients without OSCC and OPMD have obtained controversial results (30, 37). In the present study, this association was not observed. However, in Asia, where the use of chewing tobacco is more common and its use is associated with OPMD such as OSMF (25, 31), sLDH levels are higher in OSMF than in OL (29). Chewing tobacco may have a greater carcinogenic effect than cigarettes by increasing sLDH. In fact, there are studies that observed that chewing tobacco patients and patients who smoked and chewed tobacco at the same time had more micronuclei of exfoliated cells in the oral mucosa than cigarette smokers (39). Therefore, these differences in habits may have influenced the results.

In the present study, alcohol consumption, another risk factor for OSCC (2–5), was associated with higher sLDH levels than those who did not consume alcohol, but not significantly. Only one previous study observed no association between sLDH and alcohol consumption (43). Therefore, future studies in patients with OPMD and OSCC should assess this variable.

Age was positively correlated with sLDH levels, a finding consistent with previous research (41, 43). Furthermore, this variable was a significant predictor of sLDH levels. Perhaps the presence of more diseases and the use of more medications, common in older patients, may influence the sLDH values. Periodontitis influences sLDH values (41, 43–46) and is more frequent with increasing age.

Studies that have evaluated the possible association between periodontitis and OSCC have observed a weak relationship (53, 54). Although periodontitis has been observed to be associated with the prevalence of OSCC, no increased risk of OSCC has been observed in patients with periodontitis (54). Patients with OSCC usually show greater alveolar bone loss, clinical attachment loss, PPD and BOP than controls, as well as greater tooth loss (53, 54). Our study evaluated the periodontal indicators of the included patients, which has not been done in the previous studies. In the present study, OSCC patients had higher PPD and BOP values than OPMD patients and CG; and PPD was the only significant variable influencing the sLDH in the linear regression model. Other previous studies in non-OSCC/OPMD patients have observed higher sLDH values in patients with periodontitis or with higher values in its indicators (PPD, BOP, PI) than in patients with gingivitis and healthy patients (41, 43–46). sLDH levels are also reduced after ultrasonic scaling (45). Therefore, it seems of great importance to assess the periodontal status of patients when measuring this biomarker, as periodontal indicators may alter sLDH levels.

The present study is the second to evaluate sLDH in OLP patients. Gholizadeh et al. (27) observed that sLDH was not significantly higher in OLP patients than in healthy patients, while it was significantly higher in the OSCC group than in the OLP group. Therefore, our results are partly consistent with this study, as no significant differences were observed between OLP patients and CG. However, unlike us, these authors did obtain differences between OSCC and OLP. Therefore, more studies determining sLDH in OLP and OSCC patients are needed, as OLP is one of the most observed OPMD worldwide.

On the other hand, in the present study, sLDH values were higher in OLP than in OL patients, although not significantly. In the study conducted by Gholizadeh et al. (27), patients with OL were not included. Therefore, we cannot compare our results with previous studies. In addition, in the present study OLP patients with erosive and/or ulcerative lesions had higher sLDH levels than those with white lesions (reticular or plaque), although not significantly. OLP is a chronic inflammatory disorder, and it is possible that inflammation increases sLDH results.

The results of the present study suggest an association between inflammation and sLDH values, as higher sLDH levels were observed in symptomatic OLP patients and were correlated with periodontal predictors, such as PPD and BOP. These findings demonstrate that inflammation increases sLDH values and should be considered when evaluating its levels. However, inflammation has also been associated with the onset and progression of many types of cancer. Inflammation is related to immunosuppression, giving rise to a favorable microenvironment for tumorigenesis. In fact, chronic, persistent, dysregulated and untreated inflammation may be associated with cancer. Both processes, inflammation and tumorigenesis, may be present and alter biomarkers such as sLDH (55). Therefore, it is advisable to evaluate oral inflammatory processes in future studies and to take into account that if they become chronic, they could promote an environment that favors the appearance of OSCC.

Higher sLDH values were associated with higher degrees of dysplasia in patients with OL and erythroplakia. A previous study observed higher sLDH values in poorly differentiated OSCC (28). While the present study did not observe significant differences in sLDH as a function of OSCC differentiation, values were higher in poorly and moderately differentiated OSCCs than in well-differentiated OSCCs. Regarding dysplasia, the mean sLDH was lower in OL patients with dysplasia, although only 2 of the 17 patients with OL had mild dysplasia. In addition, although not previously observed, the mean sLDH levels increased progressively with increasing tumor size, being higher in OSCC cases with T4. Further studies are needed to evaluate whether sLDH can predict progression to malignancy in the case of OPMD and to predict progression and survival of OSCC patients.

The etiopathogenesis of each OPMD and OSCC is different, and this could influence the results of sLDH. Smoking and alcohol use, the main risk factors for OSCC in Europe (56), were higher in the OSCC group than in CG in the present study. OSCC patients also had the highest PPD, BOP, and age of all groups. These factors may have resulted in the highest sLDH values among all groups. The etiology of OL is unknown, but it is usually more frequent in smokers (1, 8). In fact, the number of smokers in the OL group was the highest. In our study, smoking patients had lower sLDH values. Furthermore, in the OL group, PPD, BOP and PI values were higher than in the CG, but lower than in the OSCC group. These factors could have influenced the low sLDH results in the OL group. OLP is a chronic inflammatory disease of unknown origin (8, 49). This group had the lowest percentage of smokers. However, BOP and PI were higher than in the CG. Patients with erosive or ulcerative OLP had higher sLDH levels, and these patients, experiencing pain when brushing, had a higher PI. This could have resulted in OLP patients having elevated sLDH values, behind those of OSCC, and higher than patients in the OL and CG.

sLDH levels may differ due to different habits, health practices and genetic factors between Asian and European populations. In Southeast Asia, the use of smokeless tobacco and the consumption of areca nut products is more common; however, in Europe, cigarette smoking is more used (1). Chewing tobacco has a greater carcinogenic effect (40, 56). Therefore, these differences in tobacco types could influence higher sLDH levels and a high prevalence of OSCC in Asian countries. According to WHO (2022), the prevalence of severe periodontal disease in people aged 15 years and older is 21.8% in India (57) and 6.7% in Spain (58). Periodontal markers influence sLDH levels and, therefore, these results could also be the reason for the higher levels of sLDH in Asian countries. Therefore, it is necessary to improve oral hygiene and dental care in Asian countries to reduce periodontal problems. Differences in genetic alterations could also play an important role; in fact, in Asian countries such as Pakistan, with a high prevalence of OSCC, the occurrence of GSTM1 and/or GSTT1 null genotypes together with CYP1A1 variant alleles could be the cause of increased susceptibility to OSCC (56).

Previous research (27) has observed the potential of sLDH for detecting OSCC and other OPMD, such as oral lichenoid reaction, using ROC curves. Our findings showed important dispersion and variability, with the standard deviation of the mean sLDH exceeding half the size of the mean in all cases. This high dispersion complicates the determination of cut-off values that establish a higher risk of malignancy, suggesting that, in our case, sLDH may not be a good prognostic biomarker for malignancy. Future studies should focus on establishing cut-off values to determine the risk of possible malignancy.

This study has obvious limitations due to its cross-sectional design. Prospective cohort studies are needed to assess the diagnostic validity of sLDH in OPMDs and OSCC patients. Despite a sample size calculation, the small size of each group studied limits the generalizability and statistical power of the results. Additionally, since patients were recruited from two centers in Spain, the results may not be generalizable. Multicenter observational studies involving larger and more diverse populations of OPMD and OSCC from maxillofacial surgery and oral medicine services worldwide would be desirable. These studies would help to validate the findings of the present study and to assess whether patient origin, inherent risk factors, and OPMD subtypes influence the sLDH values. Furthermore, given the potential confounding factors observed, future studies should include comprehensive data on epidemiological, clinicopathological and periodontal variables. Prospective observational studies would also be valuable to assess whether sLDH levels help predict malignancy of OPMD to OSCC.

In conclusion, although the results of the present study show higher sLDH values in OSCC and OPMD patients than in a CG, it is not possible to state that sLDH is a good predictive biomarker for malignancy progression. Future multicenter studies, including different OPMD and OSCC, would be advisable. Salivary inflammatory biomarkers, such as sLDH, may vary according to age, patient habits, and other oral and systemic processes. Improving the methodology of future studies and accurately collecting these variables is essential to identify the most reliable biomarkers for assessing the malignancy of OPMD and diagnosing OSCC. Failure to take them into account may lead to misdiagnosis.

## Data Availability

Raw data will be made available upon reasonable request from the corresponding author.
